# Case Report: Triple autoimmune overlap: rheumatoid arthritis, systemic lupus erythematosus, and hypereosinophilic asthma with systemic manifestations

**DOI:** 10.3389/fimmu.2025.1659370

**Published:** 2026-01-15

**Authors:** Ji Li, Jing Zhang, Sheng-Guang Li, Lina Zhang, Yadan Zou, Ting Long, Ruohan Yu, Yanfeng Zhang

**Affiliations:** Department of Rheumatology and Immunology, Peking University International Hospital, Beijing, China

**Keywords:** ANCA-associated vasculitis, autoimmune disease overlap, case report, eosinophilic granulomatosis with polyangiitis, hypereosinophilic asthma with systemic manifestations, immune dysregulation, rheumatoid arthritis, rhupus

## Abstract

**Background:**

Overlap between rheumatoid arthritis (RA) and systemic lupus erythematosus (SLE) (“rhupus”) is recognized, but coexistence with a severe eosinophilic asthma syndrome is exceptionally rare. We describe a triple autoimmune overlap of RA, SLE, and hypereosinophilic asthma with systemic manifestations (HASM), initially managed as ANCA-negative eosinophilic granulomatosis with polyangiitis (EGPA) and subsequently re-classified in light of evolving concepts.

**Case presentation:**

A 44-year-old woman with a 10-year history of seropositive RA developed alopecia, Coombs-positive hemolytic anemia, hypocomplementemia, and ANA and anti-Sm positivity, fulfilling SLE criteria. While receiving prednisone, hydroxychloroquine and conventional DMARDs, she subsequently developed adult-onset asthma, chronic rhinosinusitis with nasal polyps, and marked hypereosinophilia (>3.5×10^9^/L). Secondary causes were excluded; bone marrow showed reactive eosinophilia and ANCA (indirect immunofluorescence and ELISA for MPO/PR3) remained negative. She was diagnosed and treated as ANCA-negative EGPA with high-dose glucocorticoids plus methotrexate and hydroxychloroquine, leading to rapid normalization of eosinophils and durable remission of asthma and sinus disease.

**Discussion:**

In retrospect, and according to the ERS/GERM’O’P proposal, this eosinophilic disorder is best classified as HASM within the EGPA–hypereosinophilic spectrum because ANCA and biopsy-proven vasculitis were absent. The case illustrates the evolving boundary between EGPA and hypereosinophilic syndromes and extends the concept of rhupus to include an EGPA-spectrum eosinophilic asthma syndrome.

**Conclusion:**

New-onset eosinophilic asthma in patients with established rheumatic disease should prompt evaluation for EGPA-spectrum or hypereosinophilic disorders. Even when the final label is HASM rather than definite EGPA, timely institution of EGPA-type immunosuppression may avert organ damage.

## Introduction

Rheumatoid arthritis (RA) and systemic lupus erythematosus (SLE) are distinct systemic autoimmune diseases with different clinical and immunological profiles. RA is a chronic inflammatory arthritis characterized by synovial pannus formation, joint erosion, and seropositivity for rheumatoid factor (RF) and anti-citrullinated protein antibodies (ACPA), typically driven by T helper-1 (Th1) and Th17 immune responses (1). SLE is a multi-system autoimmune disease marked by loss of tolerance to nuclear antigens (e.g., ANA, anti-dsDNA, anti-Sm antibodies), immune-complex deposition with complement consumption, and a prominent type I interferon signature driving B-cell hyperactivity (2). Eosinophilic granulomatosis with polyangiitis (EGPA), formerly Churg–Strauss syndrome, is an ANCA-associated vasculitis characterized by adult-onset asthma, allergic rhinosinusitis, peripheral blood eosinophilia, and granulomatous small-vessel vasculitis affecting predominantly the respiratory tract and peripheral nerves (3). Approximately half of EGPA patients have ANCA (usually p-ANCA directed against myeloperoxidase), while the others are ANCA-negative (4). Notably, recent guidelines have proposed that ANCA-negative patients without clear vasculitis might be classified separately from “classic” EGPA (5) – an important point for this case. In the present report, we therefore interpret our patient’s eosinophilic disease as belonging to this EGPA–hypereosinophilic spectrum and will refer to it as hypereosinophilic asthma with systemic manifestations (HASM) when discussing the final diagnosis.

Overlap between RA and SLE – colloquially known as “rhupus” – was first described in 1971 (6). Patients with rhupus exhibit clinical features of both diseases (for example, erosive arthritis of RA alongside serologic and systemic features of SLE) (7). While rhupus is uncommon, it is a well-documented overlap syndrome. In contrast, overlap between either RA or SLE and an ANCA-associated vasculitis (AAV) is *rarely* reported. Each condition alone can cause some vasculitic manifestations (e.g., RA can lead to rheumatoid vasculitis, SLE can cause immune-complex vasculitis), but development of a bona fide AAV such as microscopic polyangiitis (MPA), granulomatosis with polyangiitis (GPA), or EGPA in an RA or SLE patient is unusual. Nevertheless, cases of RA overlapping with AAV have been documented as a “lesser recognized” overlap syndrome. One review identified only 35 reported cases of RA–AAV overlap up to 2015, most being RA with subsequent MPA (8). Similarly, SLE overlapping with AAV has been described in small series; for example, French cohorts reported a few patients fulfilling criteria for both SLE and AAV (mostly MPO-ANCA vasculitis) who often presented with rapidly progressive GN and pulmonary hemorrhage (9). These instances suggest such overlaps, while rare, are real and may represent distinct syndromic phenomena rather than coincidence.

**Figure 1:**
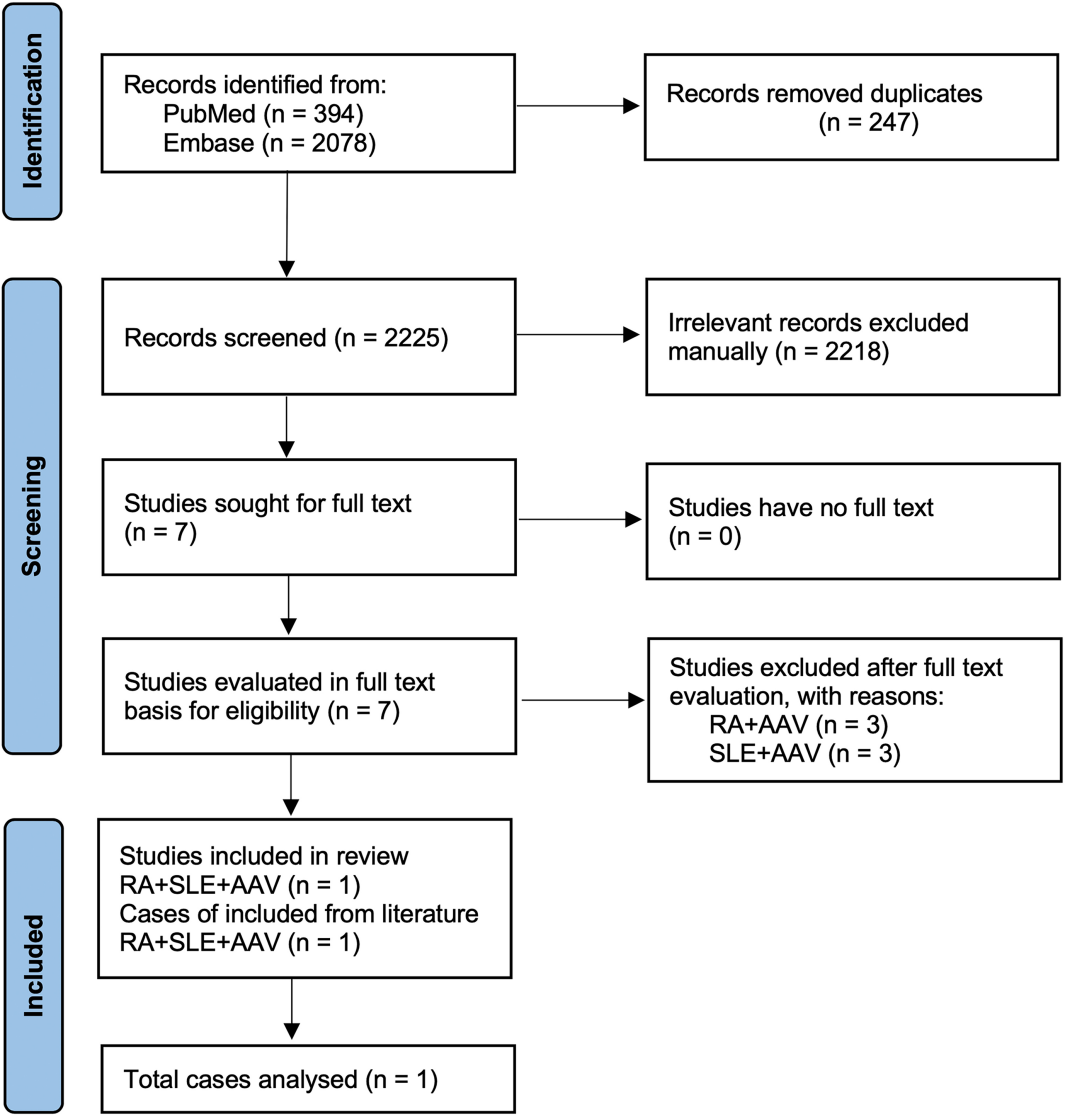
PRISMA flow diagram of the literature search for reported triple overlap syndromes involving rheumatoid arthritis (RA), systemic lupus erythematosus (SLE), and ANCA-associated vasculitis (AAV). This flowchart summarizes the systematic search and screening process conducted in PubMed and Embase to identify previously published cases reporting the coexistence of RA, SLE, and an AAV-spectrum disease. Because eosinophilic granulomatosis with polyangiitis (EGPA) is classified within AAV, AAV-related terms were used to ensure comprehensive capture of relevant EGPA-spectrum reports. From an initial 2,472 records (394 from PubMed and 2,078 from Embase), 7 articles were retrieved for full-text review after removal of duplicates and irrelevant publications. Only one study fulfilled inclusion criteria as a true triple overlap (RA+SLE+AAV), whereas the remaining six represented dual overlap syndromes (RA+AAV or SLE+AAV), underscoring the exceptional rarity of triple autoimmune overlap involving an AAV-spectrum disorder.

The combination of all three conditions – RA, SLE, and an EGPA-spectrum eosinophilic syndrome – in one patient is extraordinarily rare. In fact, a recent 2023 case report detailed what appears to be the first such *triple overlap* of RA, SLE, and AAV: that patient had MPO-ANCA-positive vasculitis consistent with MPA developing 35 years into RA/SLE (10). The authors (Reesor et al.) noted no prior literature had documented RA–SLE overlap (“rhupus”) concomitant with an AAV. To investigate further, we performed our own literature search for any reported cases of combined RA, SLE, and specifically EGPA. We reviewed over 2,400 records (PubMed and Embase through 2025) and found no published cases of a triple overlap involving EGPA. The only pertinent report was the aforementioned RA+SLE+MPA case by Reesor et al (10). This suggests our patient’s case of concurrent RA, SLE, and an EGPA-spectrum hypereosinophilic asthma with systemic manifestations is the first of its kind, highlighting an exceptionally rare overlap syndrome. **Figure 1** illustrates the literature screening process and outcome in a PRISMA flow diagram.

Given the novelty of this “triple overlap” syndrome, we herein report the case in detail and discuss the diagnostic challenges, management considerations, and potential immunological links. We also review overlapping syndromes between these diseases to contextualize our patient’s condition. Recognizing such overlaps is crucial, as new manifestations might be erroneously attributed to an existing diagnosis – timely identification of a second or third autoimmune disease can prompt appropriate therapy that might otherwise be overlooked.

## Case presentation

### Patient history

A 44-year-old woman (never-smoker) with a longstanding history of RA presented to our hospital for evaluation of new systemic symptoms. She had been well until her early 30s, when she developed polyarthritis characterized by symmetrical swelling and pain in the wrists, knees, ankles, and small joints of the hands, accompanied by prolonged morning stiffness. She was diagnosed with Rheumatoid Arthritis around 2008 (at age ~34) after tests showed a high rheumatoid factor and erosions on hand X-rays [fulfilling the 2010 ACR/EULAR RA criteria (11)]. She initially received intermittent therapy including traditional Chinese herbal medicine and occasional conventional Disease Modifying Anti-Rheumatic Drugs (DMARDs), though with inconsistent follow-up. These provided partial symptom relief but did not prevent progression. Over the years, her RA remained active; by her early 40s, she had developed severe joint deformities (including swan-neck deformities of several fingers and flexion contractures in some joints), indicating long-standing erosive disease. Despite the chronic arthritis, she had no significant systemic manifestations of RA in those years (no rash, no vasculitis, no pulmonary fibrosis). There was no family history of autoimmune diseases. She worked in an office job and had no known toxic exposures.

About three years before this presentation, in the absence of regular rheumatologic care, her arthritis worsened further. Approximately one year ago, she began experiencing new symptoms that were unusual for RA: notably, diffuse hair loss (alopecia), along with dryness of eyes and mouth (sicca symptoms). She denied any skin rashes, oral ulcers, photosensitivity, or Raynaud’s phenomenon at that time. Concerned by these developments, she empirically started a short course of prednisone 5 mg daily for two weeks (self-administered) which gave slight improvement, and then she sought evaluation at our tertiary center.

### Initial evaluation and “rhupus” diagnosis

**Figure 2:**
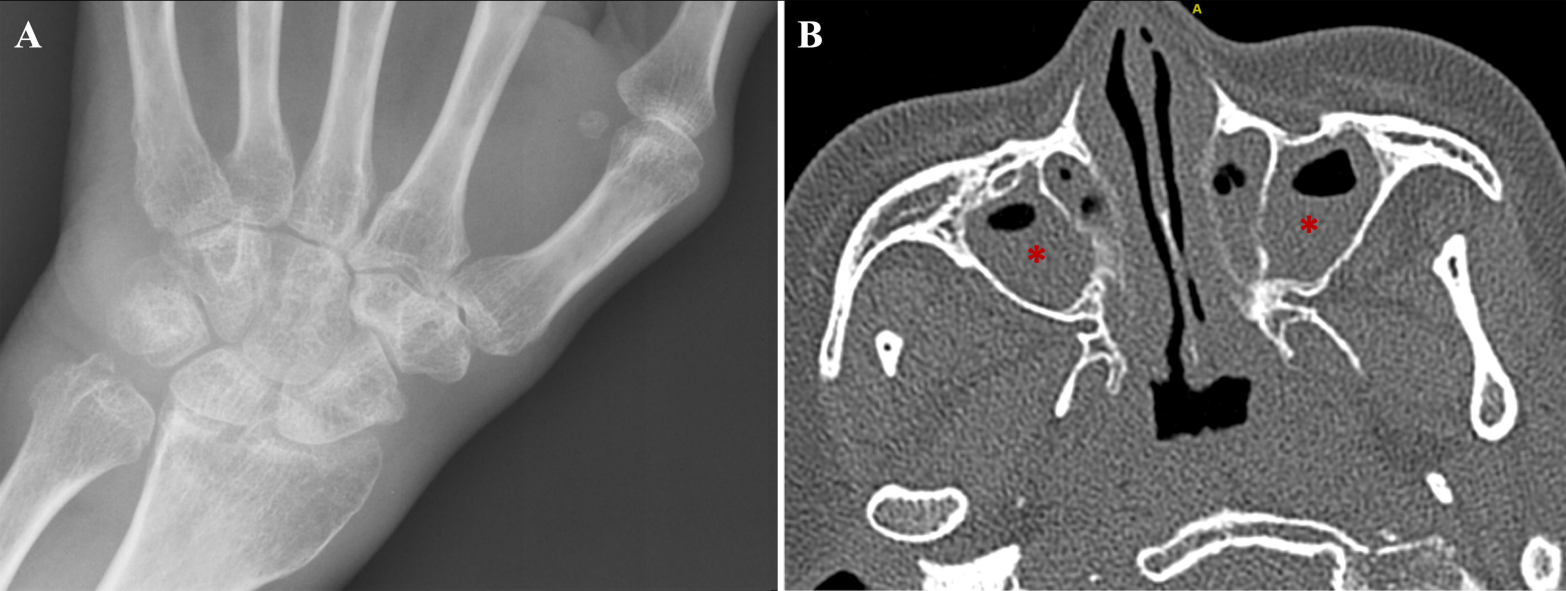
Representative imaging findings of RA and eosinophilic ENT involvement (HASM) in the patient. **(A)** Plain radiograph of the right wrist demonstrating characteristic erosive joint changes with periarticular osteopenia, consistent with longstanding rheumatoid arthritis (RA). **(B)** Axial computed tomography (CT) scan of the paranasal sinuses showing significant mucosal thickening and opacification (marked with red asterisks) in the maxillary sinuses bilaterally.

On admission to our hospital (about a year ago), the patient had active polyarthritis with synovitis in multiple joints and the aforementioned hand deformities. She also exhibited diffuse, non-scarring alopecia (hair thinning over the scalp) and complained of significant fatigue. **Table 1** summarizes the key clinical findings and timeline. Laboratory tests at that time showed elevated inflammatory markers (ESR 45 mm/h, CRP 32.8 mg/L). She had a normocytic anemia (hemoglobin 91 g/L) and a marked eosinophilia of 2.32 × 10^9^/L (27.9% of leukocytes) – an unexpected finding in an RA patient. Her rheumatoid factor was 437 U/mL and anti-CCP antibody 130.7 U/mL (both strongly positive, consistent with seropositive RA). Lupus serologies were conducted given her new alopecia and anemia: ANA was positive at 1:80 titer (homogeneous pattern), anti-SmD1 antibodies were positive, and complements were low (C3 0.84 g/L, C4 0.09 g/L; normal C3 ~0.90–1.80 g/L, C4 ~0.10–0.40 g/L). A direct antiglobulin (Coombs) test was strongly positive, indicating autoimmune hemolysis. Notably, ANCA testing was negative at that time (by IFA screening). Additional tests showed normal liver/kidney function and no evidence of infection. Imaging studies included hand X-rays (confirming erosive changes consistent with advanced RA) (**Figure 2A**) and a chest CT, which incidentally showed mild axillary lymphadenopathy and some esophageal wall thickening but no lung infiltrates or nodules. An abdominal ultrasound revealed a mildly enlarged spleen (splenomegaly).

**Table 1:** Laboratory results in the patient with rhupus and EGPA-spectrum hypereosinophilic asthma with systemic manifestations (HASM).

Laboratory parameter (units)	Reference range	Initial rhupus Dx	HASM onset	Follow-up
WBC (×10^9^/L)	4–10	8.3	8.4	7.0
Eosinophils (×10^9^/L)	0–0.5 (0–5%)	2.32 (27.9%)	3.55 (42.7%)	0.3 (4%)
Hemoglobin (g/L)	120–150	91	105	130
Platelet count (×10^9^/L)	150–400	320	280	250
ESR (mm/h)	0–20	45	12	8
CRP mg/L	0–10	32.8	3	2
ANA	Negative	Positive (1:80)	Positive (1:80)	Positive
Anti-Sm	Negative	Positive	Positive	Positive
RF (U/mL)	<20	437	437	250
Anti-CCP (IU/mL)	<17	130.7	130	80
ANCA (IIF)	Negative	Negative	Negative	Negative
ANCA (ELISA, RU/ml)	MPO & PR3, <20	Negative	Negative	Negative
C3 (g/L)	0.9–1.8	0.84	1.10	1.20
C4 (g/L)	0.1–0.4	0.09	0.20	0.30
Direct Coombs test	Negative	Positive	Positive	Negative
Reticulocyte count (%)	0.5–1.5	6.00	2.50	1.00
LDH (U/L)	140–280	400	250	200
Total bilirubin (µmol/L)	3–20	35	15	10
IgE (IU/mL)	<100	–	252	50
Allergy panel	Negative	–	Negative	–
Stool ova & parasite tests	Negative	–	Negative	–

Laboratory findings at three clinical stages: initial rhupus diagnosis, HASM onset, and follow-up. Reference ranges are provided in parentheses for clinical interpretation. ANCA was tested using both indirect immunofluorescence (IIF) and ELISA for MPO and PR3 antibodies. Complement levels (C3, C4) were considered low based on standard laboratory cutoffs. Allergy and parasitic evaluations were negative. WBC, white blood cell count; Hb, hemoglobin; ESR, erythrocyte sedimentation rate; CRP, C-reactive protein; ANA, antinuclear antibody; RF, rheumatoid factor; Anti-CCP, anti–cyclic citrullinated peptide; ANCA, anti-neutrophil cytoplasmic antibody; IIF, indirect immunofluorescence; ELISA, enzyme-linked immunosorbent assay; MPO, myeloperoxidase; PR3, proteinase 3; C3/C4, complement components 3/4; LDH, lactate dehydrogenase; IgE, immunoglobulin E.

Collectively, the patient’s presentation met classification criteria for both RA and SLE simultaneously – an overlap often referred to as “Rhupus” (6, 7). She fulfilled the 2010 ACR/EULAR RA classification criteria (11) and the 2019 EULAR/ACR SLE classification criteria (12). We diagnosed her with an RA/SLE overlap syndrome. Treatment was initiated with prednisone 30 mg daily, plus leflunomide (20 mg daily) and hydroxychloroquine (400 mg daily) as steroid-sparing agents, along with symptomatic care (calcium/vitamin D, etc.). Because of the autoimmune hemolytic anemia, she also received a short course of intravenous immunoglobulin (IVIG). Her symptoms improved modestly: the arthritis became less active, and her alopecia stabilized. Over the next few months, prednisone was tapered off successfully after the hemolysis resolved (her hemoglobin improved to ~105 g/L, and reticulocyte counts normalized; LDH and bilirubin remained within normal limits throughout, indicating that the hemolysis was well-controlled).

### Emergence of eosinophilic respiratory features

About six months after the rhupus diagnosis, the patient developed new problems. She had been maintained on leflunomide and hydroxychloroquine, but due to newly diagnosed hypertension (likely a side effect of leflunomide) (13), that drug was switched to methotrexate (MTX) 10 mg weekly. Her RA/SLE overlap disease was under relative control on this regimen (no active synovitis, and SLE serologies even showed some improvement with complement levels rising to low-normal). However, she began experiencing respiratory symptoms that she had never had before: she developed adult-onset asthma with episodes of wheezing and shortness of breath, requiring inhaled bronchodilators. She also had chronic rhinosinusitis with nasal congestion and recurrent nasal polyps causing obstruction. Additionally, she reported intense skin itching (pruritus) without any rash or obvious dermatologic lesions – she would scratch, resulting in excoriations but no urticarial lesions or vasculitic rash. These symptoms were unusual in the context of her known diagnoses. Over this period, her blood tests consistently showed persistent eosinophilia, ranging from 2.0 to 4.0 × 10^9^/L (20–45% of leukocytes on differential). This degree of eosinophilia, especially in a patient on low-dose steroids (she was on prednisone 5 mg during part of this time), was alarming.

A broad evaluation for secondary causes of eosinophilia was undertaken by her local physicians and later at our center. Parasitic infections were a chief concern: she underwent multiple stool examinations for ova/parasites and serologic tests for common parasites (including Strongyloides, schistosomiasis, filaria, etc.), all of which were negative. She empirically received a course of albendazole (400 mg daily for 3 days) for possible occult helminth infection; this had no effect on her eosinophil count or symptoms (notably, albendazole would not cover Strongyloides, but ivermectin was not given at that time). Allergy and immunology work-up was also performed: her total serum IgE was elevated (252 IU/mL; normal <100), consistent with atopy, but specific allergen testing was unremarkable. Skin prick tests for common airborne allergens and aspergillus were negative, and she did not have clinical features of allergic bronchopulmonary aspergillosis (no bronchiectasis on imaging, and aspergillus-specific IgE was not detected). We also reviewed her medications and exposures to rule out drug-induced eosinophilia – aside from her DMARDs (which are not typical causes of eosinophilia), there were no new drugs. No herbal supplements or over-the-counter medications known to trigger eosinophilia were identified. Hematologic malignancy was considered: a bone marrow biopsy was performed, which showed reactive eosinophilia with no evidence of a myeloproliferative neoplasm (there were no blasts or abnormal cells, and testing for common fusion genes associated with chronic eosinophilic leukemia – including FIP1L1-PDGFRA, ETV6-PDGFRB, and PCM1-JAK2 – was negative). These results indicated a reactive process rather than a primary blood disorder. Given her history of autoimmune disease, the constellation of adult-onset asthma, chronic sinusitis with polyposis, and unexplained eosinophilia increasingly suggested an eosinophilic granulomatous disease. Thus, she was referred back to our hospital for further management of a suspected vasculitic overlap.

### Hospital readmission and evaluation

Upon readmission (this is the current presentation), the patient’s vital signs were stable (blood pressure 136/89 mmHg on antihypertensives). She still had the chronic deformities from RA (swan-neck fingers, some mild swelling of MCP joints). She had multiple scratch marks on her skin from itching, but no palpable purpura or rash. Notably, no peripheral neuropathy was present on neurologic exam (no motor or sensory deficits, intact reflexes). Her lungs were clear to auscultation between asthmatic episodes, and heart exam was normal. There were no signs of active SLE (no mucosal ulcers or serositis). Mild splenomegaly was again noted on abdominal exam.

Laboratory tests on this admission confirmed significant eosinophilia: absolute eosinophil count 3.55 × 10^9^/L (42.7%). Her hemoglobin was 105 g/L (improved from prior, indicating the hemolysis had abated) and inflammatory markers were now normal (CRP <5 mg/L, reflecting quiescent RA/SLE). Her immunologic labs still showed positive RF and anti-CCP, as expected in RA, and ANA and anti-Sm remained positive (though dsDNA was negative; complement C3 was 0.90 g/L, C4 0.12 g/L – slightly low-normal with reference to ranges given). Importantly, ANCA was tested again and remained negative; we specifically performed both indirect immunofluorescence (which showed no c-ANCA or p-ANCA pattern) and enzyme immunoassays for anti-MPO and anti-PR3, all of which were negative. Total IgE was elevated at 252 IU/mL, consistent with her eosinophilic/allergic profile. Repeat stool tests for parasites were negative. As mentioned, bone marrow exam showed reactive eosinophilia with no malignant cells.

Imaging studies were updated. A high-resolution CT of the sinuses revealed pan-sinusitis with mucosal thickening and bilateral nasal polyps (**Figure 2B** shows an example slice). In contrast, an inspiratory chest CT showed clear lungs—no infiltrates, nodules, or ground-glass opacities. Expiratory imaging was not performed. Pulmonary function testing (July 5, 2019) showed preserved spirometric indices: FVC 3.30 L (118% predicted) and FEV1 2.65 L (111% predicted), with an FEV1/FVC ratio of 80% (99% predicted). Small-airway indices were reduced, particularly FEF25–75% 2.66 L/s (79% predicted) and FEF75% 0.93 L/s (57% predicted). Lung volumes indicated air trapping (TLC 5.44 L [124% predicted], RV 2.14 L [144% predicted], RV/TLC 39% [116% predicted]). Diffusing capacity was near normal (DLCO 19.6 mL/min/mmHg [90% predicted]). Airway resistance (Raw) was 2.92 cmH_2_O/L/s (130% predicted) (**Supplementary Table S1**). Post-bronchodilator reversibility testing and bronchial provocation testing were not performed, as these tests were not routinely available at our institution at the time of examination. A transthoracic echocardiogram was done to screen for eosinophilic cardiac disease; it was entirely normal, with normal ejection fraction and no valvular abnormalities or thrombi. We did not pursue a cardiac MRI given the normal echo and lack of cardiac symptoms. No biopsy of the nasal mucosa or lungs was obtained; given the absence of a focal lesion and the patient’s improving condition (see below), an invasive procedure was not undertaken.

### Diagnosis and treatment

The patient’s clinical presentation – adult-onset asthma, chronic rhinosinusitis with polyps, and persistent high eosinophil counts (>10% of leukocytes) in the context of existing RA/SLE – strongly suggested eosinophilic granulomatosis with polyangiitis (EGPA). She met several items of the 1990 ACR classification criteria for EGPA (asthma, eosinophilia >10%, sinusitis), although she lacked others (no neuropathy, no pulmonary infiltrates, no biopsy) (14). We considered the newer 2022 ACR/EULAR classification criteria, and her features (obstructive airway disease, nasal polyps, eosinophils >1×10^9^/L) would likely suffice for classification as EGPA (15). At that time, based on these criteria and the need for prompt treatment, we made a working diagnosis of ANCA-negative, biopsy-unproven EGPA and proceeded with therapy accordingly. Only later, during manuscript preparation and after the publication of the ERS/GERM’O’P proposal restricting the term EGPA to ANCA-positive and/or biopsy-proven vasculitic disease (16), did we re-evaluate her case and conclude that her eosinophilic syndrome fits better within hypereosinophilic asthma with systemic manifestations (HASM) on the EGPA–HES spectrum (17). In the remainder of this report, we therefore refer to this component as HASM, while acknowledging that it was initially managed as probable EGPA in routine clinical practice.

**Figure 3:**
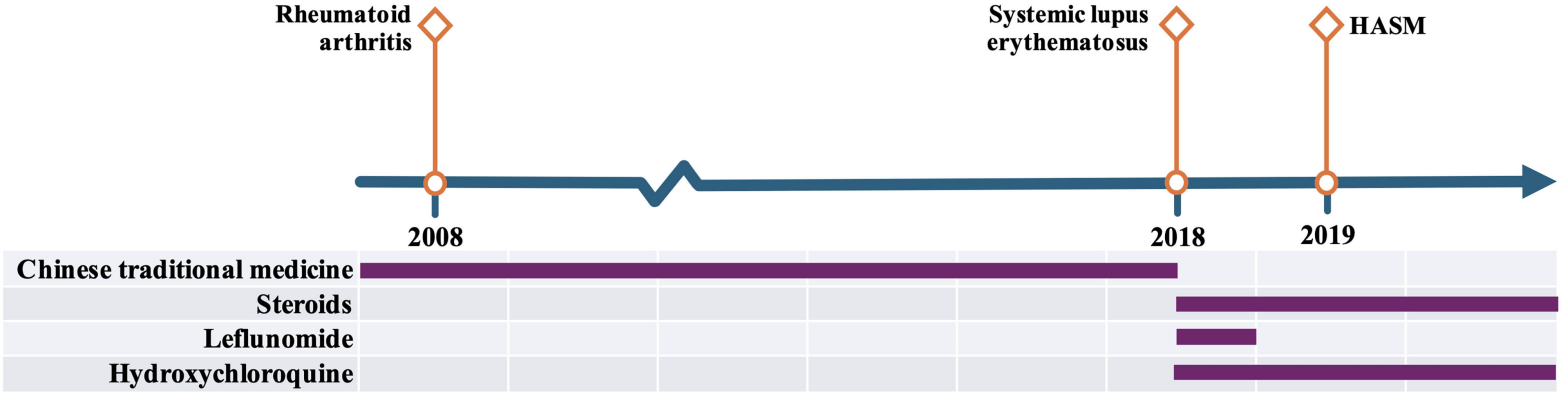
Timeline of the patient’s overlapping autoimmune syndromes and treatments. The patient was diagnosed with RA in 2008, followed by SLE in 2018, and an EGPA-spectrum hypereosinophilic asthma with systemic manifestations (HASM) in 2019, which was initially managed as ANCA-negative EGPA.

**Figure 3** illustrates the timeline of her diagnoses and treatments. We initiated high-dose corticosteroid therapy promptly. She received three days of IV methylprednisolone (1 g/day pulses) followed by oral prednisone ~50 mg daily. Her background immunosuppression with MTX (10 mg weekly) and hydroxychloroquine was continued (these would help control RA/SLE and possibly have some steroid-sparing effect). We also began topical nasal glucocorticoids for her sinus disease. Given the risk of strongyloidiasis in any patient from an endemic area receiving high-dose steroids, we considered prophylactic ivermectin; however, our patient was from a non-endemic region and had multiple negative parasite screens, so empiric anti-parasitic therapy was limited to the albendazole she had already received (in retrospect, an ivermectin course would have been prudent to cover *Strongyloides*; fortunately, she experienced no parasitic complications).

The response to treatment was excellent. Within one month, her eosinophil counts normalized (dropping to <0.5 × 10^9^/L) and her asthma and sinus symptoms resolved. The nasal polyps regressed significantly with the combination of systemic and topical steroids – by clinical examination, her nasal passages were much clearer. Her RA and SLE also remained quiescent during this period (no joint flares, and no lupus symptoms). We began a gradual taper of prednisone: by 3 months, she was at 20 mg daily; by 6 months, she reached 5 mg daily. At last follow-up (6 months after onset of the eosinophilic asthma/HASM syndrome), she was on prednisone 5 mg, MTX 10 mg weekly, and hydroxychloroquine 400 mg daily. She had no active arthritis, no respiratory symptoms, and her laboratory parameters were all within normal ranges (including complete blood count with a normal differential, and inflammatory markers remained low). A follow-up sinus CT at that time confirmed complete resolution of sinusitis and disappearance of polyps, indicating radiographic remission. Chest imaging remained normal. We planned to continue low-dose prednisone for a total of at least 12 months from the onset of the EGPA-spectrum eosinophilic asthma/HASM, given the relapsing nature of EGPA, and then consider tapering off completely if she remained stable. We also discussed the potential need for steroid-sparing strategies (such as adding an IL-5 inhibitor like mepolizumab or using rituximab which could address both vasculitis and RA) if she had difficulty tapering or if any component of her disease relapsed. To date, however, she continues to do well.

This case therefore represents a patient with sequential development of RA, then SLE, then an EGPA-spectrum eosinophilic asthma syndrome (HASM) over roughly a decade. For clarity, we present a timeline of her disease course in **Figure 3**.

## Discussion

We describe what appears to be the first documented case of rhupus (RA–SLE overlap) complicated by an EGPA-spectrum hypereosinophilic asthma with systemic manifestations (HASM). Clinically, at the time of presentation, the eosinophilic component was managed as ANCA-negative EGPA; however, in light of more recent ERS/GERM’O’P Task Force proposal (17), we now classify it as HASM within the hypereosinophilic syndrome spectrum. By the end of her disease course, our patient had clear clinical evidence of three autoimmune processes – RA, SLE and HASM. This case expands the spectrum of known overlap syndromes in rheumatology and underscores the importance of maintaining a broad differential when new symptoms arise in patients with established autoimmune diseases. This conservative approach is supported by recent recommendations that reserve the term EGPA for cases with ANCA or histologic vasculitis (5). In practice, however, the distinction may be academic – our patient required aggressive immunosuppressive therapy for her eosinophilic disease regardless of terminology.

### Overlap in context

Overlap syndromes involving two autoimmune diseases are recognized (albeit uncommon) but overlap of three is exceptional. As noted, overlapping RA and SLE (rhupus) has been well-documented since the 1970s (18, 19). RA overlapping with AAV is rare; most reported cases have been RA coexisting with MPA or GPA, often with severe manifestations like renal disease (8, 20, 21). SLE overlapping with AAV is similarly infrequent (9, 22, 23), sometimes presenting as an ANCA-positive lupus nephritis that essentially behaves like an AAV. Our patient’s course was unusual in that her eosinophilic component manifested with eosinophil-rich respiratory tract involvement (asthma, sinusitis) rather than renal or skin vasculitis. EGPA typically features a Th2-driven eosinophilic inflammation of the airways and often is ANCA-negative (8). This contrasts with GPA or MPA, which are more often Th1/Th17-driven, affect kidneys/lungs/ENT with granulomatous inflammation, and are usually ANCA-positive. The absence of renal involvement in our case is consistent with the pattern more often seen in ANCA-negative EGPA, in which cardiopulmonary and ENT manifestations predominate, and renal disease occurs only in a minority of cases (24). However, because our patient remained ANCA-negative and never developed biopsy-proven vasculitis, we now interpret these features as an EGPA-spectrum hypereosinophilic asthma with systemic manifestations (HASM) rather than definite EGPA. We did not observe any neuropathy, which is another common EGPA feature (often seen in ANCA-positive cases); its absence may reflect early intervention or simply the particular expression of EGPA-spectrum eosinophilic disease in this patient.

From a diagnostic standpoint, our case raises the question of how to classify patients like hers. As mentioned, the 2022 ACR/EULAR classification criteria for EGPA assign weighted points to features such as asthma, eosinophil count ≥1 × 10^9^/L, neuropathy, nasal polyps, pulmonary infiltrates, etc., with ≥6 points needed to classify EGPA (15). These criteria were developed using data from the Diagnostic and Classification Criteria in Vasculitis (DCVAS) study and have high sensitivity and specificity. If we apply her data: she gets points for asthma, eosinophils >1 × 10^9^/L, nasal polyps, and maybe others (she’d likely reach the threshold of 6). Thus, she qualifies as EGPA by those criteria. However, it must be emphasized that classification criteria are meant for research and assume the patient is already suspected of having a vasculitis. They are not a substitute for clinical diagnosis. Clinically, one must rule out other causes of eosinophilia and ideally obtain a biopsy to confirm vasculitis before pronouncing EGPA. In our patient, despite exhaustive evaluation, we ended up without a tissue diagnosis.

### Diagnostic evolution——from EGPA to HASM

Clinically, we initially classified the eosinophilic component of this overlap syndrome as EGPA because the patient fulfilled traditional clinical definitions (adult-onset asthma, eosinophilia >10%, and sino-nasal disease) and reached the threshold of the 2022 ACR/EULAR classification score (15). However, she remained ANCA-negative and never developed clear vasculitic manifestations such as neuropathy, renal involvement, skin purpura, or biopsy-proven small-vessel vasculitis. In parallel, the ERS/GERM’O’P Task Force has proposed that patients with severe eosinophilic asthma, blood hypereosinophilia and systemic symptoms but without ANCA or documented vasculitis should be labelled as “hypereosinophilic asthma with systemic manifestations (HASM)” within the hypereosinophilic syndrome spectrum rather than EGPA (5). In this context, we now regard our patient as having rhupus complicated by HASM, which was managed according to EGPA-spectrum recommendations. We believe that this diagnostic re-evaluation is educational, as it illustrates how evolving classification systems can change nomenclature without substantially altering bedside management in real-world practice.

Cardiac involvement is a key determinant of prognosis in EGPA-spectrum disease, and myocarditis may be clinically silent. Cardiac magnetic resonance (CMR) provides tissue characterization and is considered the reference non-invasive imaging modality for diagnosing myocarditis (including eosinophilic myocarditis), whereas echocardiography may remain normal in early disease (25). Therefore, CMR should be considered in EGPA-spectrum patients when there is clinical suspicion or to evaluate for subclinical myocardial involvement. In our patient, CMR was not performed; thus, subclinical myocardial involvement cannot be fully excluded.

### Differential diagnosis

In evaluating our patient, we carefully excluded alternative diagnoses that can present with hypereosinophilia and systemic features. One major consideration was idiopathic hypereosinophilic syndrome (HES) (26). HES is defined by persistent eosinophilia ≥1.5 × 10^9^/L for >6 months causing organ damage, after excluding secondary causes. Our patient certainly had high eosinophils and some organ involvement (lungs, sinuses), but HES is typically a diagnosis of exclusion. We excluded secondary causes (no parasite, no allergy, no malignancy). The line between ANCA-negative EGPA and HES can be blurry; some experts consider EGPA a subset of hypereosinophilic disorders when vasculitis is absent (as in our patient). What pointed us toward EGPA was the triad of asthma, sinusitis, and eosinophilia, which is classic for EGPA and not for other HES variants. Furthermore, her bone marrow showed reactive eosinophilia, and genetic tests for primary eosinophilic leukemia were negative, which fits with a secondary (likely autoimmune) eosinophilia.

We also considered IgG4-related disease (IgG4-RD), since it can cause asthma, elevated IgE, and even eosinophilia in some cases (27). However, IgG4-RD usually presents with tumor-like organ involvement (e.g., pancreatitis, salivary gland swelling, orbital pseudotumor) and high serum IgG4 levels (28). Our patient had none of these features – no organ enlargement on imaging, and her serum IgG4 (which we checked) was not elevated. Drug-induced eosinophilia was another differential; we scrutinized her medications but found no culprit. Neither MTX, HCQ, nor leflunomide are known to cause hypereosinophilia or pulmonary eosinophilia. She was not on antibiotics or other drugs that commonly trigger eosinophilic pneumonia. Thus, a drug reaction (such as DRESS or a chronic drug-induced eosinophilic syndrome) was unlikely.

Another important consideration was allergic bronchopulmonary aspergillosis (ABPA), an asthma-associated eosinophilic lung disease caused by hypersensitivity to *Aspergillus*. ABPA can present with severe asthma, pulmonary infiltrates, bronchiectasis, and markedly elevated IgE levels (29). However, our patient had no pulmonary infiltrates or bronchiectasis on imaging, and her total IgE was not significantly elevated, making ABPA unlikely. Similarly, chronic eosinophilic pneumonia (CEP) can cause pulmonary infiltrates and peripheral eosinophilia, but our patient had no lung infiltrates, and her eosinophilia was accompanied by systemic features beyond the lungs, so CEP was not a fit.

Finally, we rigorously pursued parasitic infections and treated empirically. Especially relevant is *Strongyloides stercoralis*, which can cause asymptomatic eosinophilia and then hyperinfection if steroids are given (30). We tested for *Strongyloides* serologically (it was negative) but—acknowledging imperfect sensitivity—we would normally treat empirically with ivermectin prior to high-dose steroids. In our patient’s case, because all tests were negative and her exposure risk was low, we started steroids after giving albendazole (which, as noted, is inadequate for *Strongyloides*). In retrospect, a 2-day ivermectin course would have been ideal; fortunately, the patient did not develop any issues suggestive of an undiagnosed parasitic infection. In summary, none of these alternative etiologies accounted for her eosinophilic illness, and critically, no evidence of vasculitis was found on workup. These findings support that her eosinophilic disease represents a non-vasculitic process (HASM) rather than a classic EGPA.

### Immunologic considerations and therapeutic implications

The sequential development of RA, then SLE, then an EGPA-spectrum eosinophilic asthma syndrome (HASM) in a single individual suggests a broad underlying immune dysregulation with the capacity to shift phenotypes over time. RA is classically a Th1/Th17-driven disease at the pathological level (pro-inflammatory cytokines like TNFα, IL-17, etc., mediate synovitis), whereas SLE is characterized by autoantibody production and immune-complex deposition facilitated by B cells and plasmacytoid dendritic cell interferon production (31). EGPA, in contrast, is the prototypical Th2-dominant disease: patients have high levels of IL-5, IL-4, IL-13 which drive eosinophil expansion and activation (32). It is remarkable – and in our view, instructive – that our patient’s immune system traversed this entire spectrum: from a Th1/Th17-mediated arthritis (RA) to a B-cell and interferon-driven systemic autoimmunity (SLE), to a Th2-driven eosinophilic disease on the EGPA–HES spectrum. One hypothesis is that she has an underlying genetic predisposition that broadly elevates her risk for autoimmunity; for example, polymorphisms in PTPN22 and CTLA4 are known to increase the risk of multiple autoimmune diseases, including RA, SLE, and ANCA-associated vasculitis (10). Such factors might have set the stage, while environmental exposures or even the immunomodulatory effects of medications could have tipped the immune balance at various points (for instance, it is speculated that certain immunosuppressants might skew cytokine profiles over time). However, this remains speculative – our case does not provide mechanistic proof, but it raises interesting questions.

Another common thread across RA, SLE, and EGPA is the role of B cells. Autoreactive B cells produce RF and anti-CCP in RA, a wide array of autoantibodies in SLE, and in EGPA, B cells can produce ANCA (when present) and interact with eosinophils. The success of B-cell depletion therapy (rituximab) in treating RA and AAV suggests a pathogenic role for B cells in both. Indeed, in previously reported overlap cases (including one RA–Churg–Strauss overlap), rituximab was effective in inducing remission when other therapies failed (20). In the RA–SLE–MPA case by Reesor et al., rituximab plus steroids was used to treat the vasculitis successfully (10). In our patient, we achieved remission with corticosteroids (plus MTX); she has not required rituximab to date. But if she were to relapse or develop refractory disease, targeting B cells or IL-5 could be logical therapeutic strategies given the immunologic milieu.

Managing a patient with three overlapping autoimmune conditions requires a nuanced approach. One must address all components of the disease without over-immunosuppressing the patient unnecessarily. In our case, we needed to control the eosinophilic inflammation (with its potential for organ-threatening vasculitic complications) while also maintaining suppression of RA and SLE to prevent flare-ups. Fortunately, corticosteroids are effective for all three diseases and were the cornerstone of therapy during the eosinophilic asthma/HASM phase. We maintained her on methotrexate and hydroxychloroquine, which likely helped prevent flares of RA and SLE (and methotrexate might also have some steroid-sparing effect on EGPA). Notably, although our induction approach mirrored that for EGPA (high-dose glucocorticoids plus methotrexate), the absence of any confirmed vasculitis or ANCA meant that the patient’s illness is ultimately classified as a non-vasculitic hypereosinophilic asthma with systemic manifestations (HASM) rather than true EGPA.

We avoided cyclophosphamide or other cytotoxic drugs because she did not have life-threatening organ involvement (no cardiac or renal manifestations of EGPA) – high-dose steroids were sufficient. This tailored approach is in line with recommendations that severe AAV (e.g., EGPA with heart or neuropathy involvement) might warrant cyclophosphamide or rituximab, whereas limited disease can be managed with glucocorticoids and conventional immunosuppressants (33, 34). Several diagnostic evaluations were not available or not pursued in this case. Post-bronchodilator reversibility testing and bronchial provocation testing were not performed because these tests were not routinely available at our institution at the time, which limits a formal assessment of airway reversibility. In addition, cardiac magnetic resonance was not performed; therefore, subclinical myocardial involvement cannot be completely excluded despite a normal echocardiogram. Close monitoring is essential; we continue to follow her for any sign of relapse in any of her disease components. Thus far, the overlap of RA/SLE has stayed in remission, which we attribute to ongoing therapy, and the HASM/EGPA-spectrum component has not recurred after tapering steroids to a low dose.

From a broader perspective, cases of polyautoimmunity like this serve as “natural experiments” to explore common pathways in autoimmunity. There is evidence that certain individuals are predisposed to multiple autoimmune diseases, supporting the concept of a shared autoimmune diathesis (10). Studying such patients could reveal biomarkers or gene expression changes as their disease phenotype shifts – for example, one could investigate whether our patient showed a transition from a Th1/Th17 signature to a Th2 signature over time. Such research, while outside the scope of this report, could deepen our understanding of the interplay between different autoimmune conditions.

## Conclusion

In summary, we have reported an extremely rare case of a patient with coexisting RA, SLE, and an EGPA-spectrum hypereosinophilic asthma with systemic manifestations (HASM), initially managed as ANCA-negative EGPA. This case illustrates that even in patients with a known autoimmune disease, clinicians must remain alert to the emergence of additional autoimmune or eosinophilic processes when new, unexplained symptoms arise. Early recognition of such an overlap – in this instance, identifying an EGPA-spectrum hypereosinophilic asthma in a patient with prior rhupus – is critical to initiate appropriate therapy before significant organ damage occurs. Our patient’s course also highlights shared immunological threads that link disparate rheumatic diseases, from genetic risk factors to the central role of B cells and cytokine networks. While definitive classification of ANCA-negative eosinophilic disease remains debated, we believe that reframing this case as HASM, rather than definite EGPA, is most consistent with current proposals and may help refine how similar patients are described and managed in the future.

## Data Availability

The original contributions presented in the study are included in the article/**Supplementary Material**. Further inquiries can be directed to the corresponding author.

## Supplementary material

Supplementary Table 1 Pulmonary function test results (July 5, 2019). Values are reported as measured baseline (pre-bronchodilator) results with corresponding predicted (reference) values and percent predicted where available. Post-bronchodilator reversibility testing and bronchial provocation testing were not performed because these tests were not routinely available at our institution at the time of examination. %Pred, percent predicted; DLCO, diffusing capacity of the lung for carbon monoxide; DLCO/VA, transfer coefficient (DLCO normalized to alveolar volume); DL Adj, hemoglobin-adjusted DLCO (as reported); FEV1, forced expiratory volume in 1 second; FEF25–75%, forced mid-expiratory flow; FEF75%, forced expiratory flow at 75% of FVC; FVC, forced vital capacity; PEF, peak expiratory flow; Raw, airway resistance; RV, residual volume; TLC, total lung capacity; VA, alveolar volume; VC, vital capacity.
